# Medically Treated Nonischemic Thin-Cap Fibroatheroma Lesions Versus Fractional Flow Reserve-Guided Complete Revascularization in Diabetic Patients

**DOI:** 10.1016/j.jscai.2023.101256

**Published:** 2023-12-12

**Authors:** Tobias M. Hommels, Renicus S. Hermanides, Enrico Fabris, Krzysztof P. Malinowski, Balázs Berta, Tomasz Roleder, Fernando Alfonso, Giuseppe De Luca, Rohit M. Oemrawsingh, Wojciech Wojakowski, Arnoud W.J. van ‘t Hof, Elvin Kedhi

**Affiliations:** a Isala Hartcentrum, Isala Hospital, Zwolle, the Netherlands; b Cardiovascular Department, University of Trieste, Trieste, Italy; c Department of Bioinformatics and Telemedicine, Faculty of Medicine, Jagiellonian University Medical College, Kraków, Poland; d Center for Digital Medicine and Robotics, Jagiellonian University Medical College, Kraków, Poland; e Heart and Vascular Center, Semmelweis University, Budapest, Hungary; f Department of Cardiology, Regional Specialist Hospital, Wrocław, Poland; g Department of Cardiology, Hospital Universitario La Princesa, Madrid, Spain; h Department of Cardiology, AOU Maggiore della Carità, Eastern Piedmont University, Novara, Italy; i Department of Cardiology, Albert Schweitzer Hospital, Dordrecht, the Netherlands; j Division of Cardiology and Structural Heart Diseases, Medical University of Silesia, Katowice, Poland; k Department of Cardiology, Maastricht University Medical Center, Maastricht, the Netherlands; l Department of Cardiology, Zuyderland Medical Center, Heerlen, the Netherlands; m Department of Cardiology, McGill University Health Center, Montreal, QC, Canada; n Department of Cardiology, Hôpital Erasme, Université libre de Bruxelles, Brussels, Belgium

**Keywords:** diabetes mellitus, fractional flow reserve, optical coherence tomography, percutaneous coronary intervention, thin-cap fibroatheroma

## Abstract

**Background:**

Fractional flow reserve (FFR) is an established method to guide decisions on revascularization; however, in patients with diabetes mellitus (DM), FFR-negative lesions carrying an optical coherence tomography-detected thin-cap fibroatheroma (TCFA) remain at high risk for adverse cardiac events.

**Methods:**

In this prespecified subanalysis of the COMBINE OCT-FFR trial, DM patients with ≥1 FFR-negative, TCFA-positive medically treated target lesions referred to as vulnerable plaque (VP group), were compared to patients with exclusively FFR-positive target lesions who underwent complete revascularization (CR group). The primary endpoint was first and recurrent event analysis for target lesion failure and the secondary endpoint was a composite of cardiac death, target vessel myocardial infarction, target lesion revascularization, or hospitalization due to unstable angina.

**Results:**

Among 550 patients enrolled, 98 belonged to the VP group while 93 to the CR group and were followed up to 5 years. The VP group had a higher occurrence of the primary endpoint (20.4% vs 8.6%; HR, 2.22; 95% CI, 0.98-5.04; *P* = .06). Recurrent event analysis showed that the VP group had significantly higher rates of the primary and secondary endpoints (9.17 vs 3.76 events per 100 PY; RR, 2.44; 95% CI, 1.16-5.60; *P* = .01 and 13.45 vs 5.63 events per 100 PY; RR, 2.39; 95% CI, 1.30-4.62; *P* < .01).

**Conclusions:**

In a population with DM, medically treated nonischemic, TCFA-carrying target lesions were associated with higher risk of reoccurring adverse cardiac events compared to target lesions that underwent complete revascularization, opening the discussion about whether a focal preventive revascularization strategy could be contemplated for highly vulnerable lesions.

## Central Illustration

**Figure:**
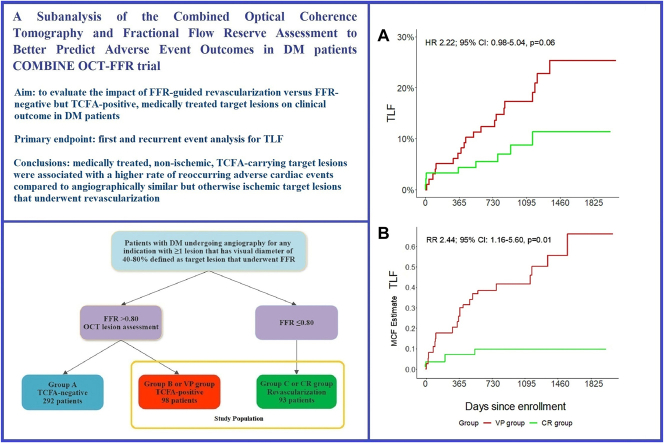

## Introduction

Fractional flow reserve (FFR) is a well-established modality to determine ischemic lesions in coronary artery disease that may benefit from revascularization.^1^^,^^2^ However, recent trials have brought to question the validity of an ischemia-guided revascularization in the setting of stable coronary artery disease as well as acute coronary syndrome (ACS).^3^, ^4^, ^5^ Furthermore, these trials corroborate what is previously observed in nonrandomized studies which have shown that in certain proinflammatory and prothrombotic conditions, like diabetes mellitus (DM) and ACS, deferring revascularization on the basis of FFR guidance is associated with higher rates of adverse cardiac events.^6^, ^7^, ^8^, ^9^ Conversely, the impact of plaque morphology and composition is emerging as a strong predictor of future events even in absence of ischemia. In particular, vulnerable plaque such as lesions containing a thin-cap fibroatheroma (TCFA) has been identified as a very strong predictor of future adverse cardiac events.^9^, ^10^, ^11^ It has been postulated that TCFA-carrying lesions are dynamic in nature and associated with rapid plaque progression as a consequence of silent plaque disruption and subsequent healing.^12^^,^^13^ Furthermore, the development of high-resolution imaging modalities like optical coherence tomography (OCT) has facilitated TCFA detection. Moreover, a recently published substudy from our team showed that OCT-detected, lipid-rich TCFA, rather than any lipid-rich plaque as previously believed, is strongly associated with future adverse events.^14^ Two ongoing large randomized trials, the COMBINE-INTERVENE (NCT05333068) and INTERCLIMA (NCT05027984), are testing the hypothesis if an OCT-detected vulnerable plaque-guided revascularization, either alone or in combination with FFR, is superior to revascularization by FFR guidance alone. As shown in the Central Illustration, this prespecified analysis from the COMBINE OCT-FFR trial compares the outcomes of DM patients with medically treated, nonischemic, TCFA-positive lesions with DM patients who had exclusively ischemic lesions and underwent complete revascularization based on FFR guidance alone. This comparison is of interest as it indirectly evaluates the impact of revascularization as compared to optimal medical treatment for morphologically vulnerable and angiographically intermediate to severe but otherwise nonischemic lesions.

**Central Illustration:**
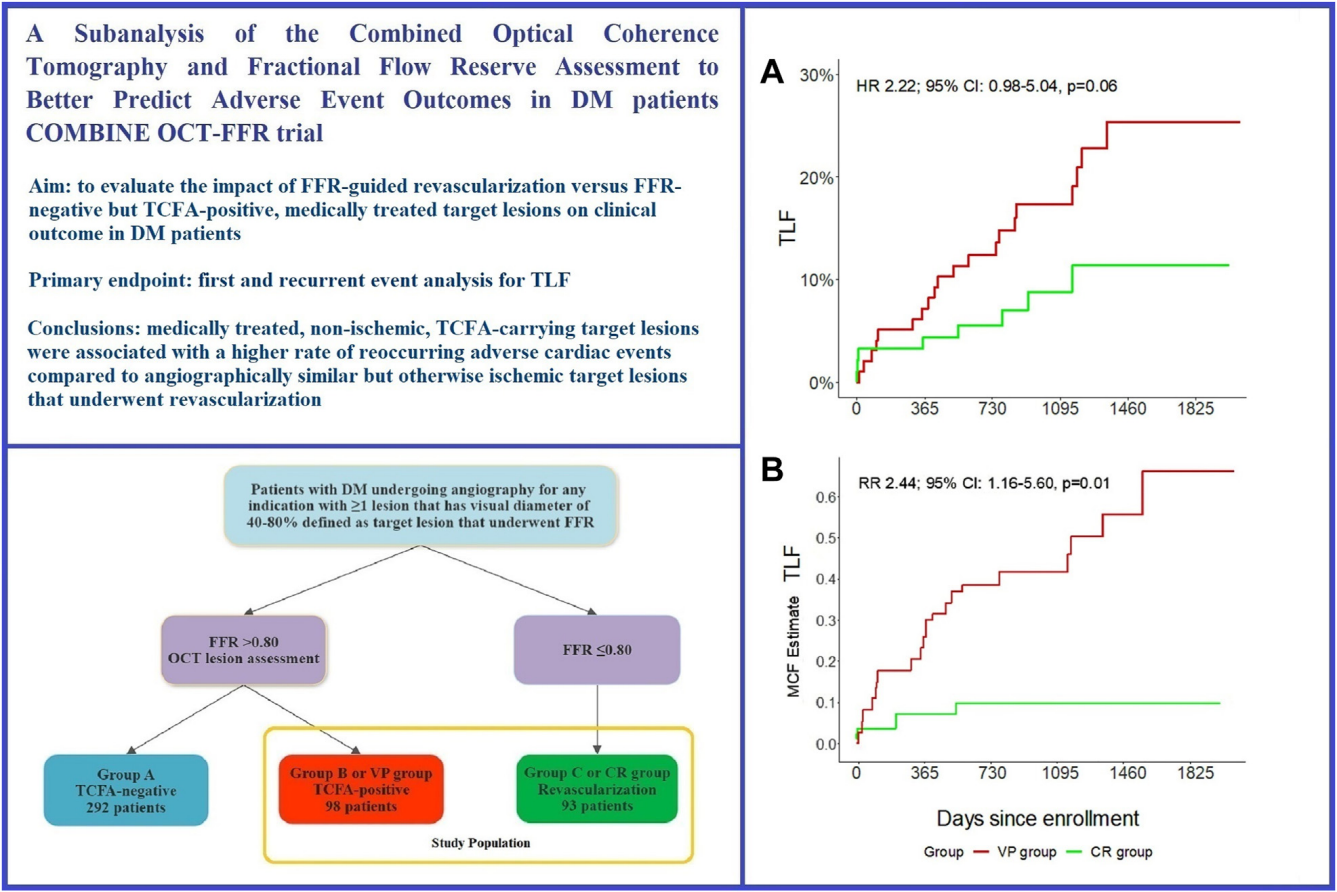
Outcomes of medically treated nonischemic thin-cap fibroatheroma lesions versus FFR-guided complete revascularization in diabetic patients. Shown is the overview of a subanalysis of the COMBINE OCT-FFR trial in which the outcomes of DM patients with nonischemic, vulnerable plaques (VP group) were compared to DM patients who underwent complete FFR-guided revascularization (CR group). The primary endpoint was TLF. (**A**) In first-time event analysis, the VP group had a higher occurrence of TLF. (**B**) In recurrent event analysis, presented as MCF indicating the number of target lesion-orientated events a patient could experience at the designated time of follow-up, the VP group had a statistically significant higher occurrence of TLF. DM, diabetes mellitus; FFR, fractional flow reserve; HR, hazard ratio; MCF, mean cumulative function; OCT, optical coherence tomography; RR, rate ratio; TCFA, thin-cap fibroatheroma; TLF, target lesion failure.

## Methods

The present is a prespecified analysis from the COMBINE OCT-FFR trial. The COMBINE OCT-FFR (NTR5376/NCT02989740) was a prospective, double-blind, international, natural-history study. The design and study protocol have been reported previously.^15^

### Study population and procedure

In brief, DM patients who underwent coronary angiography for either stable coronary artery disease or ACS were eligible for inclusion if ≥1 target lesion was present. Target lesions were defined as de novo, native, nonculprit coronary lesions with visual stenosis of 40% to 80%. In patients presenting with ACS, the culprit lesion was treated first. All target lesions underwent FFR assessment. If FFR was positive (≤0.80), mandatory revascularization was performed. If FFR was negative (>0.80), an OCT assessment of the vessel was conducted. Both the operators and treated patients were blinded for the outcomes of the OCT procedures. Patients with ≥1 FFR-negative target lesion(s) were further divided into 2 groups based on absence or presence of a TCFA, respectively Group A and B. Patients with exclusively positive FFR (≤0.80) target lesions who underwent revascularization of all lesions represent Group C. All patients received optimal medical therapy according to the guidelines. All adverse cardiac events were adjudicated by an independent clinical event committee. Patients were considered diabetic if they received insulin or oral hypoglycemic regimens. For the purpose of this substudy, we compared patients with FFR-negative (>0.80) and TCFA-positive target lesions referred to as vulnerable plaque (VP group, originally Group B) to patients that underwent complete revascularization based exclusively on positive FFR values (≤0.80) in all target lesions (CR group, originally Group C).

The OCT analysis was based on the consensus standards for acquisition, measurement, and reporting of intravascular optical coherence tomography studies of the American College of Cardiology.^16^ The image analysis consisted of serial cross-sectional images starting 5 mm distal to 5 mm proximal on OCT-defined borders of the target lesion. Signal-rich homogeneous plaques were classified as fibrous, signal-poor regions with diffuse borders as lipid-rich plaques, and signal-poor regions with well-defined borders as calcified plaques. A TCFA was defined as any coronary target lesion with a predominantly lipid-rich plaque in which the thinnest part of the atheroma cap measured ≤65 μm and lipid arc >90°. The OCT analysis was performed by 2 investigators. The interrater and intrarater agreement for TCFA detection was determined by kappa values and showed a κ = 0.81 (95% CI, 0.70-0.97) and κ = 0.78 (95% CI, 0.61-0.92), respectively. The analysis was conducted by using CAAS IntraVascular 2.0 software (Pie Medical Imaging BV).

### Study endpoints

In order to better understand the impact of FFR-negative, TCFA lesions on future outcomes, a target lesion-oriented endpoint (in this study target lesions were all lesions that underwent FFR +/– OCT assessment) was defined. The primary endpoint was target lesion failure (TLF), a composite of cardiac death, target vessel myocardial infarction (TV-MI), or target lesion revascularization (TLR). TLR was clinically driven. To better capture the urgent hospitalizations for unstable angina, a secondary endpoint was established, a composite endpoint of cardiac death, TV-MI, TLR, or hospitalization due to unstable angina. Furthermore, to provide more insights into safety outcomes, we also analyzed the rates of periprocedural and spontaneous MI and TV-MI in which the latter is defined as any MI or TV-MI that occurred >48 hours after index procedure. All endpoints were defined according to the clinical data standards of the American College of Cardiology/American Heart Association and the Academic Research Consortium.^17^

### Statistical analysis

Categorical variables are presented as frequencies and percentages. A 2-sided Pearson χ^2^ test or Fisher exact test was calculated to determine statistically significant differences between the VP group and CR group at baseline characteristics. Continuous variables with normal distribution are presented as mean and standard deviation. A *t* test was performed to determine significant differences in these variables. The primary endpoint TLF as well as the composites were analyzed using univariable Cox regression, reported as hazard ratio with 95% CI, and plotted as Kaplan-Meier estimates. Considering the highly dynamic nature of TCFA-carrying lesions and the different lengths of follow-up patients received, we also performed an estimation of recurrent event rate in patient-years for the primary and secondary endpoints as well as the composites. We used the Prentice-Williams-Peterson model with a rate ratio accompanied by the 95% CI and mean cumulative function to estimate the number of events a certain included patient could experience at a specific time point at follow-up. In addition, we performed a multivariable analysis with adjustment for the following variables: sex, age, previous ACS, current ACS, current or previous smoker, and insulin usage at baseline for both combined endpoints. The variables processed into the models were selected for correcting apparent differences in baseline characteristics between treatment groups and were selected based on associated effects on the endpoints. The selected variables were limited by the total number of events. C-index was used as a measure of goodness of fit for the multivariable models while validation was performed using bootstrap resampling. Variance inflation factors were examined to assess the multicollinearity; Schoenfeld residuals were examined to check model assumptions. A *P* value < .05 was upheld as statistically significant. Statistical analysis was performed using R 4.1.1 (The R Foundation for Statistical Computing, 2021) with packages “rms” version 6.2-0, “reda” 5.3.0, and “reReg” 1.4.0 as well as SPSS version 26 (IBM Corp).

## Results

### Patients characteristics

A total of 191 patients were included in the analysis, 98 were enrolled in the VP group and 93 in the CR group. The median follow-up was 1096 days. Baseline clinical and angiographic characteristics are shown in Table 1. Both groups showed similarities for most characteristics concerning cardiovascular risk factors and medical history. Patients in the CR group were more frequently male, while patients from the VP group had higher proportion of insulin-dependent DM patients. Clinical syndrome at presentation was similar between both groups. The angiographic characteristics at baseline showed that patients in the VP group had more lesions per patient, while the CR group had 2 times more lesions that underwent revascularization. However, the number of remaining target lesions per treatment group was similar.

**Table 1:** Baseline clinical and angiographic characteristics.

	VP group (n = 98)	CR group (n = 93)	*P* value
Age, y	67.3 ± 10.9	66.7 ± 9.4	.59
Male sex	65 (66.3)	74 (79.6)	.04
Body mass index, kg/m^2^	29.8 ± 5.0	29.4 ± 4.1	.90
Creatinine level, μmol/L	77.8 ± 35.6	68.4 ± 39.7	.36
HbA1c level, mmol/mol	57.7 ± 11.7	59.9 ± 14.2	.70
Insulin prescription at baseline	35 (35.7)	22 (23.7)	.07
Arterial hypertension	75 (76.5)	67 (72.8)	.56
Hypercholesterolemia	61 (62.2)	63 (68.5)	.37
Smoking status			
Current smoker	22 (22.5)	15 (16.5)	.30
Previous smoker	23 (34.9)	33 (44.0)	.27
Medical history			
Previous ACS	42 (42.9)	42 (45.2)	.75
Previous MI	40.8 (40)	41.3 (38)	.95
Previous PCI	41 (41.8)	43 (46.2)	.54
Previous CABG	4 (4.1)	1 (1.1)	.37
Previous CVA	12 (12.4)	5 (5.4)	.10
Clinical presentation			.09
ACS	21 (21.4)	30 (32.3)	
Non-ACS	77 (78.6)	63 (67.7)	
ACS specified			.89
ST-elevation MI	3 (14.3)	3 (10.0)	
Non–ST-elevation MI	9 (42.9)	14 (46.7)	
Unstable angina	9 (42.9)	13 (43.3)	
Angiographic characteristics	204 Lesions	150 Lesions	
Lesions per patient	2.1 ± 0.96	1.6 ± 0.77	<.01
Lesions treated	81	150	
Lesions treated per patient	0.8 ± 0.81	1.6 ± 0.77	<.01
Vessel disease			<.01
1	38 (38.8)	56 (60.2)	
2	49 (50.0)	35 (37.6)	
3	11 (11.2)	2 (2.2)	
Target lesions	123	104	
FFR-negative target lesions	123	0	
Target lesions per patient	1.26 ± 0.66	1.12 ± 0.46	.10
Distribution target lesions			<.01
Right coronary artery	44 (35.8)	17 (16.3)	
Left main	1 (0.8)	0 (0.0)	
Left anterior descending artery	45 (36.6)	75 (72.1)	
Circumflex artery	33 (26.8)	12 (11.5)	
Visual diameter stenosis, %	52.7 ± 10.9	63.0 ± 9.9	<.01

Values are mean ± SD or n (%).

ACS, acute coronary syndrome; CABG, coronary artery bypass grafting; CVA, cerebrovascular accident; FFR, fractional flow reserve; MI, myocardial infarction; PCI, percutaneous coronary intervention; UA, unstable angina.

### Outcomes

**Figure 1:**
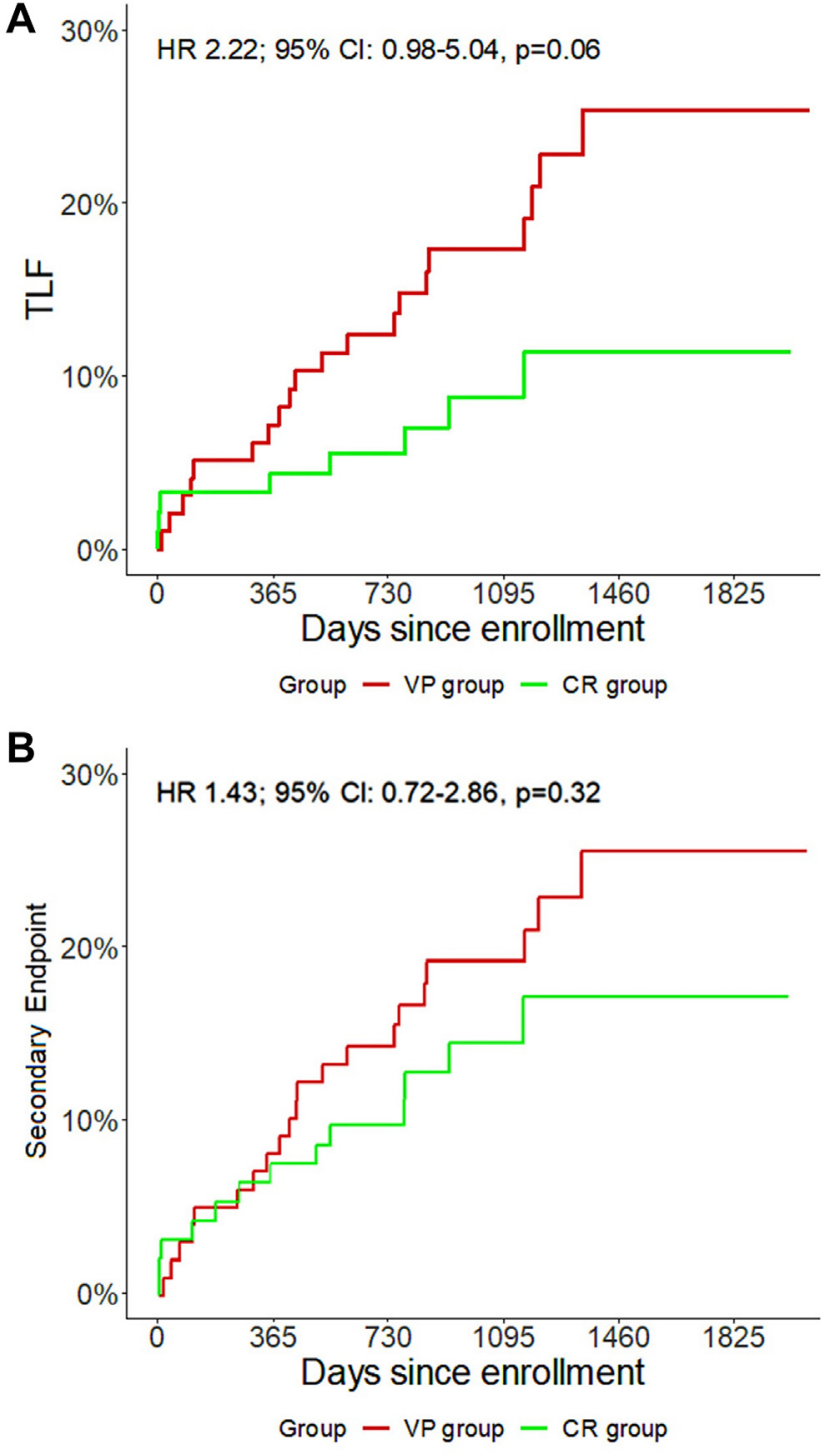
**Outcome of the primary and secondary endpoints.** Shown are Kaplan-Meier plots for the (**A**) primary endpoint target lesion failure composed of cardiac death, target vessel myocardial infarction or target lesion revascularization, and (**B**) the secondary endpoint composed of cardiac death, target vessel myocardial infarction, target lesion revascularization or hospitalization due to unstable angina. The red line represents the VP (fractional flow reserve-negative, thin-cap fibroatheroma-positive target lesions) group while the green line corresponds to the CR (completely revascularized fractional flow reserve-positive target lesions) group. HR, hazard ratio; TLF, target lesion failure.

**Figure 2:**
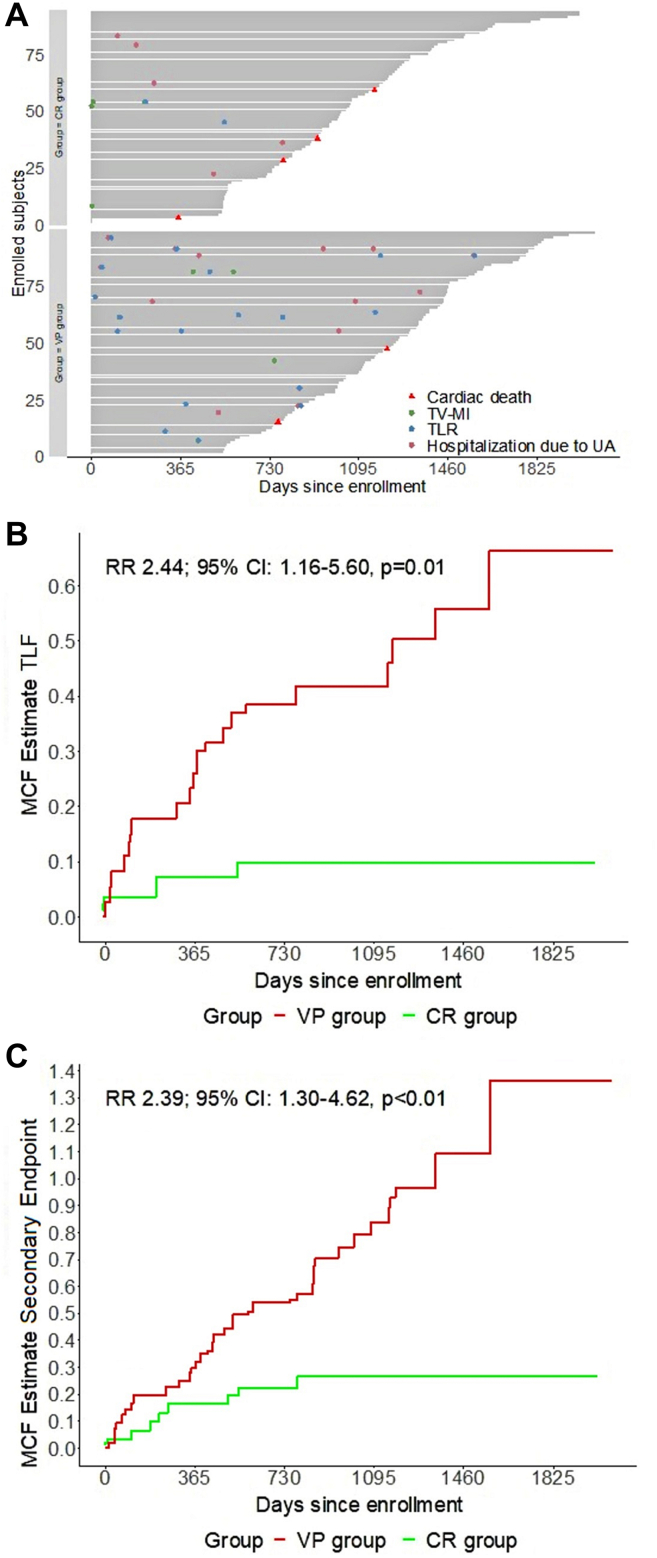
**Recurrent survival analysis for the primary and secondary endpoints.** Shown are the recurrent event incidences for (**A**) the primary and secondary endpoints in the VP (fractional flow reserve-negative, thin-cap fibroatheroma-positive target lesions) group and the CR (completely revascularized fractional flow reserve-positive target lesions) group. Shown is the mean cumulative function estimate, in which the vertical axis indicates the estimated number of events a certain patient could experience at a specific time point at follow-up, for (**B**) the primary endpoint and (**C**) the secondary endpoint. The primary endpoint was target lesion failure, a composite of cardiac death, target vessel myocardial infarction, or target lesion revascularization. The secondary endpoint was a composite of cardiac death, target vessel myocardial infarction, target lesion revascularization, or hospitalization due to unstable angina. MCF, mean cumulative function; RR, rate ratio; TLR, target lesion revascularization; TV-MI, target vessel myocardial infarction; UA, unstable angina.

The VP group as compared to the CR group showed a higher occurrence of the primary endpoint TLF (20.4% vs 8.6%; HR, 2.22; 95% CI, 0.98-5.04; *P* = .06) as shown in Table 2 and Figure 1. Furthermore, regarding the single composites of this endpoint, a significant difference was observed for TLR (17.4% vs 2.2%; HR, 7.89; 95% CI, 1.82-34.17; *P* < .01). A nonsignificant but otherwise increasingly divergent trend between both groups was reported for respectively any myocardial infarction (MI) (11.2% vs 7.5%; HR, 1.45; 95% CI, 0.56-3.73; *P* = .45), TV-MI (6.1% vs 3.3%; HR, 1.79; 95% CI, 0.45-7.16; *P* = .42) and spontaneous TV-MI (6.1% vs 1.1%; HR, 5.44; 95% CI, 0.65-45.17; *P* = .12). The secondary endpoint was not significant (21.4% vs 14.0%; HR, 1.43; 95% CI, 0.72-2.86; *P* = .32). As shown in Table 3, the recurrent event analysis showed that patients in the VP group had a significant 2.5 times higher rate of both the primary and secondary endpoints (9.17 vs 3.76 events per 100 PY; RR, 2.44; 95% CI, 1.16-5.60; *P* = .01 and 13.45 vs 5.63 events per 100 PY; RR, 2.39; 95% CI, 1.30-4.62; *P* < .01). The mean cumulative function estimator graphic for recurrent events is presented in Figure 2. Multivariable analysis identified the presence of an FFR-negative, TCFA-positive target lesion to be a predictor of adverse cardiac events for TLF (HR, 2.34; 95% CI, 0.88-6.23; *P* = .09) and independent predictor for the secondary endpoint (HR, 2.32; 95% CI, 1.24-4.34; *P* < .01) as is presented in Table 4.

**Table 2:** Results up to 5-year follow-up.

Endpoints and clinical events	VP group (n = 98)	CR group (n = 93)	Univariable Cox regression	
			HR (95% CI)	*P* value
Primary endpoint (TLF)	20.4 (20)	8.6 (8)	2.22 (0.98-5.04)	.06
Secondary endpoint^a^	21.4 (21)	14 (13)	1.43 (0.72-2.86)	.32
Cardiac death	2.0 (2)	4 (4.3)	0.37 (0.07-2.03)	.25
Any MI	11.2 (11)	7.5 (7)	1.45 (0.56-3.73)	.45
Spontaneous MI	10.2 (10)	6.5 (6)	1.55 (0.56-4.26)	.40
TV-MI	6.1 (6)	3.3 (3)	1.79 (0.45-7.16)	.42
Spontaneous TV-MI	6.1 (6)	1.1 (1)	5.44 (0.65-45.17)	.12
Any revascularization	27.6 (27)	9.7 (9)	2.85 (1.34-6.07)	<.01
Target lesion revascularization	17.4 (17)	2.2 (2)	7.89 (1.82-34.17)	<.01
Hospitalization due to UA	10.2 (10)	5.4 (5)	1.74 (0.59-2.51)	.32

Values are n (%) unless otherwise specified. Clinical outcomes represented as the primary and secondary endpoints up to 5-year follow-up are shown. The results are presented by total number of events with corresponding percentages and are also reported in univariable Cox regression analysis with 95% CI.

HR, hazard ratio; MI, myocardial infarction; TLF, target lesion failure; TV-MI, target vessel myocardial infarction; UA, unstable angina.

a The secondary endpoint was a composite of cardiac death, target vessel myocardial infarction, target lesion revascularization, or hospitalization due to unstable angina.

**Table 3:** Recurrent event results up to 5-year follow-up.

Endpoints and clinical events	VP group (n = 98)		CR group (n = 93)		RR (95% CI)	*P* value
	n	100 PY (95% CI)	n	100 PY (95% CI)		
Primary endpoint (TLF)	30	9.17 (6.19-13.09)	10	3.76 (1.80-6.91)	2.44 (1.16-5.60)	.01
Secondary endpoint^a^	44	13.45 (9.77-18.06)	15	5.63 (3.15-9.29)	2.39 (1.30-4.62)	<.01
Cardiac death	2	0.61 (0.07-2.21)	4	1.50 (0.41-3.85)	0.41 (0.04-2.84)	.41
Any MI	13	3.97 (2.12-6.80)	10	3.76 (1.80-6.91)	1.06 (0.43-2.70)	.99
Spontaneous MI	12	3.67 (1.90-6.41)	7	2.63 (1.06-5.42)	1.39 (0.51-4.18)	.65
TV-MI	7	2.14 (0.86-4.41)	4	1.50 (0.41-3.85)	1.42 (0.36-6.63)	.74
Spontaneous TV-MI	7	2.14 (0.86-4.41)	1	0.38 (0.01-2.09)	5.70 (0.73-256.72)	.08
Any revascularization	38	11.62 (8.22-15.94)	9	3.38 (1.55-6.42)	3.44 (1.63-8.08)	<.01
TLR	21	6.42 (3.97-9.81)	2	0.75 (0.09-2.71)	8.54 (2.09-75.17)	<.01
Hospitalization due to UA	14	4.28 (2.34-7.18)	5	1.88 (0.61-4.38)	2.28 (0.78-8.08)	.11

Clinical outcomes represented as the primary and secondary endpoints and clinical events up to 5-year follow-up are shown. The results are presented by recurrent event analysis in which the total number of events are reported with corresponding events per 100 patient-years (PY) with addition of 95% CI.

MI, myocardial infarction; RR, rate ratio; TLF, target lesion failure; TLR, target lesion revascularization; TV-MI, target vessel myocardial infarction; UA, unstable angina.

a The secondary endpoint was a composite of cardiac death, target vessel myocardial infarction, target lesion revascularization, or hospitalization due to unstable angina.

**Table 4:** Multivariable Cox regression analysis.

Primary endpoint (TLF)	HR (95% CI)	*P* value
FFR(–) TCFA(+) lesion	2.34 (0.88-6.23)	.09
Male sex	0.6 (0.27-1.36)	.22
Age	1.07 (1.0-1.12)	<.01
Previous ACS	1.02 (0.48-2.18)	.95
Current ACS	2.31 (1.06-5.03)	.03
Current or previous smoker	2.16 (0.92-5.07)	.08
Insulin baseline	1.22 (0.51-2.87)	.66

Secondary endpoint^a^	HR (95% CI)	*P* value
FFR(–) TCFA(+) lesion	2.32 (1.24-4.34)	<.01
Male sex	0.93 (0.49-1.77)	.93
Age	1.03 (1.00-1.07)	.05
Previous ACS	1.03 (0.60-1.76)	.93
Current ACS	1.03 (0.55-1.92)	.93
Current or previous smoker	1.44 (0.81-2.57)	.21
Insulin at baseline	1.62 (0.85-3.11)	.15

Multivariable Cox regression analysis is shown. The number of variables incorporated into the models was related to the occurred total event rates. The variables processed into the models were selected for correcting apparent differences in baseline characteristics for more homologous treatment groups and were selected for associated effects on the endpoints. A *P* value of <.05 was considered statistically significant.

ACS, acute coronary syndrome; FFR, fractional flow reserve; HR, hazard ratio; TCFA, thin-cap fibroatheroma; TLF, target lesion failure.

a The secondary endpoint was a composite of cardiac death, target vessel myocardial infarction, target lesion revascularization, or hospitalization due to unstable angina.

## Discussion

This prespecified subanalysis of the COMBINE OCT-FFR trial aimed to compare the outcomes of DM patients with coronary artery disease who underwent revascularization for all FFR-positive lesions and received further medical treatment for the remaining vulnerable TCFA-carrying target lesions versus DM patients who had only ischemic target lesions (FFR-positive) who underwent complete revascularization.

The finding of this subanalysis is that DM patients carrying medically treated, nonischemic, TCFA-containing target lesions were associated with an increase in the rate of adverse cardiac events as compared to DM patients who underwent complete revascularization. Furthermore, the CR group was associated with a significant reduction in adverse cardiac events when a recurrent event analysis was implemented capturing the detrimental dynamic nature of TCFA-carrying lesions. Multivariable analysis confirmed that the presence of an FFR-negative, TCFA-positive target lesion was, albeit not formally statistically significant for TLF, an independent predictor of worse outcomes. Importantly, these unfavorable outcomes were driven not only by TLR but also by spontaneous TV-MI, with both event rates showing a similar hazard ratio although it did not reach significance for TV-MI mainly because this study was underpowered for this particular endpoint. One of the most important mechanisms for progression of atherosclerosis is the continuous rupture and healing of vulnerable plaques.^12^^,^^13^ A plaque rupture could lead to an ACS or be completely asymptomatic. However, the subsequent healing of these ruptured plaques could lead to further reduction of coronary lumen which in turn could be associated with angina leading to revascularization. Our findings further corroborate this hypothesis as about half of the primary endpoint events in the VP arm are due to cardiac death and incidences of TV-MI while the other half is due to target lesion revascularization standalone. Derived from these results, medically treated patients that carry at least one nonischemic lesion, with a diameter stenosis between 40% and 80% containing a TCFA are associated with worse prognosis when compared to lesions that were angiographically similar but underwent revascularization based on positive FFR measurements.

Our findings are in concurrence with the results from a post hoc analysis of the 3V-FFR-FRIENDS study, in which a similar difference was observed in medically treated patients carrying a CT-detected vulnerable plaque as compared to patients who underwent revascularization based on positive FFR measurements.^8^ Interestingly, the recently published PECTUS trial further corroborated the concept already proved by the original COMBINE OCT-FFR trial that FFR-negative target lesions carrying a vulnerable plaque are associated with higher event rates during follow-up as compared to target lesions without vulnerability features.^18^ Furthermore, these findings are also in line with the findings of the diabetic subanalysis from the PROSPECT trial which showed that the majority of events during follow-up originated from the medically treated lesions rather than lesions that underwent revascularization.^9^

The PROSPECT study and other trials included only ACS patients, while the COMBINE OCT-FFR study enrolled patients with and without ACS. As the main focus was to investigate the prognosis of FFR-negative target lesions in DM patients independently from the presentation, considering that DM is intrinsically an important risk factor comparable to ACS, we do not believe that this choice of design has impacted the results. On the contrary, despite the CR arm having a higher number of patients presenting with ACS, this group still had a trend toward better clinical outcomes.

In this present subanalysis, both treatment arms showed similar baseline clinical characteristics. There was a tendency for more insulin prescriptions in patients with FFR-negative, TCFA-positive target lesions. Although prescription of insulin may indicate more severe DM, the HbA1c levels were quite similar between the arms and therefore we believe this disparity is unlikely to have significantly impacted the observed outcomes.

In this subanalysis, we observed a numerically higher rate of cardiac death in the CR group. The reason behind this could be that most cardiac deaths in DM patients events are not clearly related to the lesions studied, defined as target lesions in our study, but could be related to culprit lesions or previously treated lesions as well as such events might not be related to coronary pathology at all.

As expected, due to the nature of group selection, the angiographic data differed somehow between the groups. While there were more lesions observed in the VP group, the number of lesions revascularized in the CR group was twice as high and was generally associated with favorable outcomes underlining the good performance of modern drug-eluting stents. The results of the target lesion level endpoints were equally favorable, indirectly suggesting that a focal plaque sealing strategy, although more aggressive, could be beneficial in the treatment of TCFA-carrying lesions. This approach was already tested in the PROSPECT ABSORB trial, in which nonobstructive lesions with high-lipid burden that underwent PCI with implantation of bioresorbable scaffolds showed favorable long-term angiographic results when compared to standalone medical therapy.^19^

Conversely, while one could argue that medical treatment is sufficient for certain lesions with very high vulnerability features, most of the remaining (ischemic but nonvulnerable) lesions could be successfully treated medically, as showed in the ISCHEMIA trial.^3^^,^^4^ Indeed, only 20% of patients in the medically treated arm of this trial required a cross-over to the PCI arm due to unbearable angina. Interestingly, another important lesson from the ISCHEMIA trial was that the observed lower rate of spontaneous MI in the ischemia-guided revascularization arm was limited to 30%, suggesting that the remaining 70% could derive from lesions that did not undergo revascularization due to the absence of ischemia. Indeed, as previously described, an FFR-guided approach leads to revascularization of only 30% of the assessed lesions while the remaining 70% of the lesions, including those with high vulnerability features, are left on medical treatment and are therefore prone to future events as shown from this actual subanalysis.^8^ Altogether, this evidence suggests that a revascularization strategy that combines the treatment of severely ischemic lesions as well as vulnerable lesions could lead to further improvements in clinical outcomes as compared to ischemia guidance alone. In the future, the ongoing COMBINE-INTERVENE (NCT05333068) and INTERCLIMA (NCT05027984) randomized trials will give further insights into the validity of this approach.

The results of this subanalysis have to be interpreted taking into account different limitations related to the nonrandomized nature of the COMBINE OCT-FFR trial and the relatively small size of this subanalysis. Furthermore, the results of this study are pertinent to DM patients and should not be generalized to all-comer patients. Although prespecified, with results showing a clear trend toward a higher rate of adverse cardiac events in the VP group, this subanalysis was not sufficiently powered, and therefore the results should be considered as hypothesis generating. Although the target lesions were angiographically similar, the morphology of the target lesions treated in the CR group is unknown and may differ from that of the VP group. This study did not incorporate the impact of novel lipid-lowering and antidiabetic drugs that have shown increased efficiency in reducing future cardiovascular events.

## Conclusions

In patients with DM, medically treated FFR-negative, TCFA-carrying target lesions were associated with a higher rate of reoccurring adverse cardiac events when compared to angiographically similar but otherwise ischemic target lesions that underwent revascularization. Given the high risk of vulnerable plaques and the good performance of modern drug-eluting stents, this analysis prompts further research to evaluate whether a revascularization strategy in these highly vulnerable lesions might be associated with favorable outcomes as compared to optimal medical treatment. This finding should be regarded as hypothesis-generating and needs further corroboration from ongoing large, randomized trials.
